# Alpha-Glucosidase and Alpha-Amylase Inhibitory Activities, Molecular Docking, and Antioxidant Capacities of *Salvia aurita* Constituents

**DOI:** 10.3390/antiox9111149

**Published:** 2020-11-19

**Authors:** Ninon G. E. R. Etsassala, Jelili A. Badmus, Jeanine L. Marnewick, Emmanuel I. Iwuoha, Felix Nchu, Ahmed A. Hussein

**Affiliations:** 1 Department of Horticultural Sciences, Cape Peninsula University of Technology, Symphony Rd., Bellville 7535, South Africa; nchuf@cput.ac.za; 2 Applied Microbial and Health Biotechnology Institute, Cape Peninsula University of Technology, Symphony Rd., Bellville 7535, South Africa; marnewickj@cput.ac.za; 3 Chemistry Department, University of the Western Cape, Private Bag X17, Bellville 7535, South Africa; eiwuoha@uwc.ac.za; 4 Chemistry Department, Cape Peninsula University of Technology, Symphony Rd., Bellville 7535, South Africa; mohammedam@cput.ac.za

**Keywords:** diabetes mellitus, oxidative stress, alpha-glucosidase, alpha-amylase, *Salvia aurita*, abietane diterpenes

## Abstract

Diabetes mellitus (DM) is one of the most dangerous metabolic diseases with a high rate of mortality worldwide. It is well known that insulin resistance and deficiency in insulin production from pancreatic β-cells are the main characteristics of DM. Due to the detrimental side effects of the current treatment, there is a considerable need to develop new effective antidiabetic drugs, especially alpha-glucosidase and alpha-amylase inhibitors with lesser adverse effects. These inhibitors are known to be directly involved in the delay of carbohydrate digestion, resulting in a reduction of glucose absorption rate and, consequently, reducing the postprandial rise of plasma glucose, which can reduce the risk of long-term diabetes complications. Furthermore, natural products are well-known sources for the discovery of new bioactive compounds that can serve as scaffolds for drug discovery, including that of new antidiabetic drugs. The phytochemical investigation of *Salvia aurita* collected from Hogobach Pass, Eastern Cape Province, South Africa (SA), yielded four known abietane diterpenes namely carnosol (**1**), rosmanol (**2**), 7-methoxyrosmanol (**3**), 12-methoxycarnosic acid (**4**), and one flavonoid named 4,7-dimethylapigenin (**5**). Structural characterization of these isolated compounds was conducted using 1 and 2D NMR, in comparison with reported spectroscopic data. These compounds are reported for the first time from *S. aurita*. The biological evaluation of the isolated compound against alpha-glucosidase exhibited strong inhibitory activities for **3** and **2** with the half maximal inhibitory concentration (IC_50_) values of 4.2 ± 0.7 and 16.4 ± 1.1 µg/mL respectively, while **4** and **1** demonstrated strong alpha-amylase inhibitory activity amongst the isolated compounds with IC_50_ values of 16.2 ± 0.3 and 19.8 ± 1.4 µg/mL. Molecular docking analysis confirms the strong inhibitory activity of **3** against alpha-glucosidase. Additionally, excellent antioxidant capacities were displayed by **2**, **1,** and **3,** respectively, with oxygen radical absorbance capacity (ORAC) (25.79 ± 0.01; 23.96 ± 0.01; 23.94 ± 0.02) mM Trolox equivalent (TE)/g; **1** and **2** as ferric-ion reducing antioxidant power (FRAP) (3.92 ± 0.002; 1.52 ± 0.002) mM ascorbic acid equivalent (AAE)/g; **5** and **2** as Trolox equivalent absorbance capacity (TEAC) (3.19 ± 0.003; 2.06 ± 0.003) mM TE/g. The methanolic extract of *S. aurita* is a rich source of abietane diterpenes with excellent antioxidant and antidiabetic activities that can be useful to modulate oxidative stress and might possibly be excellent candidates for the management of diabetes. This is the first scientific report on the phytochemical isolation and biological evaluation of the alpha-glucosidase and alpha-amylase inhibitory activities of *Salvia aurita*.

## 1. Introduction

Diabetes mellitus (DM) is one of the most dangerous metabolic diseases with a high rate of mortality worldwide [1,2]. It is characterized either by a shortage in insulin production or by the degradation of produced insulin. Among the well-known existing types of DM, types 1 and 2 are the most predominant. Treatment of type 1 DM demands insulin injection, while type 2 DM requires the usage of diet control, physical exercises, and utilization of synthetic antidiabetic drugs [3]. Type 2 DM, which is mainly provoked by the degradation of produced insulin, affects more people in contrast with other types of diabetes, and patients do not rely on exogenous insulin for the prevention of ketonuria as well as ketosis [4]. The pathogenesis in type 2 diabetes is identified by the fact that the pancreas manufactures insulin, but it is not utilized correctly by the body [4], which is basically caused by peripheral tissue insulin resistance, whereby insulin receptors or other intermediates in the insulin signaling pathways inside the cells are not sensitive to insulin [5]. It results in the inability of glucose to go into the tissue, causing hyperglycemia or uplifted level of glucose concentrations in blood [6]. Obesity is one of the main menaces of type 2 diabetes, which generally results in impaired insulin action, and most patients in this case are obese [4].

Unfortunately, up to now, no cure for diabetes is available, but it can be controlled by a proper management of blood sugar levels via the consumption of a healthy diet, physical exercises, and usage of multiple synthetic antidiabetic agents, which can reduce the risk of long-term diabetes complications [7]. However, the effectiveness of these synthetic antidiabetic agents is limited because of detrimental adverse effects including flatulence, diarrhea, stomach ache, hypoglycemia, damage of liver, drug resistance, gaining weight, and heart disorder coupled with the high cost of drugs [8,9]. Therefore, there is a significant need for producing natural antidiabetic products/drugs with a high safety margin. Medicinal plants and natural products have been employed as sources of medicine since ancient times for alleviating human suffering [10], and plants are well known to be the principal source of health-promoting secondary metabolites including terpenoids, flavonoids, polyphenols, and several other valuable constituents, which are responsible for several health promoting effects such as antidiabetic activity [11].

Carnosol (**1**) was isolated for the first time from *Salvia carnosa* in 1942, and its chemical structure was elucidated in 1964 [12]. It has been reported to possess a wide range of biological activities including antidiabetic and antioxidant capabilities [13,14,15]. Rosmanol (**2**) was isolated from *Rosmarinus officinalis* L. and *Salvia chamelaeagnea* [15,16]. It has been reported to possess a strong antioxidant capacity [13,14,15]. Moreover, 7-methoxyrosmanol (**3**) was isolated from *Rosmarinus officinalis* L. [17] and has been reported to have significant activity on the central nervous system due to its ability to bind to the benzodiazepine receptor [18]. Similarly, 12-methoxycarnosic acid (**4**) was isolated from *Rosmarinus officinalis* L., *Salvia microphylla,* and *Salvia officinalis* [17,19] and possesses antimicrobial activity against *Staphylococcus aureus* [18]. Furthermore, 4,7-dimethylapigenin ether (**5**) was isolated from *Blumea balsamifera* and was reported to possess antifungal activity [20].

*Salvia aurita* (Lamiaceae, *Salvia*), commonly known as African blue sage, is an herbaceous perennial shrub native to South Africa. It is widely distributed in the Cape floristic region, KwaZulu-Natal, and Swaziland, where it grows up to 1.2 m (3.9 ft) tall on streambanks [21]. *S. aurita* has pharmaceutical properties, such as antioxidant, antiplasmodial, antimicrobial, anti-inflammatory, antituberculosis, and cytotoxicity [21]. These properties can be attributed to their secondary metabolite contents. For example, numerous *Salvia* species such as *S.* *chloroleuca*, S. acetabulosa, S. moorcroftiana, S. miltiorrhiza, S. africana-lutea, and *S. virgata* have been reported to be rich sources of phenolic compounds with prominent alpha-glucosidase and alpha-amylase inhibitory activities [9,22].

This is the first scientific study to be carried out on the phytochemical isolation and biological investigation of *S. aurita*. The findings suggest that these compounds might possibly become prominent natural candidates to inhibit alpha-glucosidase and alpha-amylase as well as oxidative stress related to diabetes with the prospect to be used in the formulation of diabetes drugs upon further biological studies.

## 2. Materials and Methods

### 2.1. Reagents

EGCG (epigallocatechin gallate), Trolox (6-hydroxyl-2,5,7,8-tetramethylchroman-2-carboxylic acid), and other reagents such as TPTZ (2,4,6-tri[2-pyridyl]-s-triazine, iron (III) chloride hexahydrate, ABTS (2,2-azino-bis (3-ethylbenzo thiazoline-6-sulfonic acid) diammonium salt), AAPH (2,2-Azobis (2-methylpropionamidine) dihydrochloride), fluorescein sodium salt, potassium peroxodisulfate, copper sulfate, hydrogen peroxide, perchloric acid, alpha-glucosidase (*Saccharomyces cerevisiae*), alpha-amylase (procaine pancreas), and 3,5,di-nitro salicylic acid (DNS), sodium carbonate (Na_2_CO_3_), 4-nitro-phenyl-α-d-glucopyranoside (pNPG), di-sodium hydrogen phosphate, and sodium dihydrogen phosphate purchased from Sigma-Aldrich, Cape Town, South Africa. The organic solvents used in this study were supplied by Merck, Cape Town, South Africa. The NMR spectra were carried out on an Avance 400 MHz spectrometer (Bruker, Rheinstetten, Germany) using deuterated chloroform. Preparative HPLC was utilized for the purification of compounds using HPLC-grade methanol and distilled water.

### 2.2. Plant Material

The plant material (aerial part) of *S. aurita* used in this study was collected in June 2017 in Eastern Cape, Province, South Africa. The identification of a voucher specimen was done by Prof. Christopher Cupido and deposited at Kirstenbosch (Compton Herbarium) with the herbarium number NBG1465541-0.

### 2.3. Extraction and Purification of Chemical Constituents

The fresh plant material (855.1 g) of *S. aurita* was grounded and extracted with 2.5 L of methanol at ambient temperature (25 °C) for 48 h. The methanol extract was filtered and then evaporated to dryness under reduced pressure using a rotary evaporator at 40 °C to produce 64.62 g (5.6%). The crude extract (62 g) was subjected to a silica gel column (25 × 18 cm) and eluted using a gradient of hexane and ethyl acetate in order of raising polarity. Sixty-two fractions were collected and combined based on their thin layer chromatography (TLC) similarities to produce 17 main fractions labeled I–XVII.

Main fraction XIII (1040 mg) was applied to repeated silica gel column chromatography using hexane and ethyl acetate in order of rising polarity ((from Hex [70%]:EtOAc [30%] to 100% EtOAc)), followed by sephadex (95% methanol). Sub-fraction XIII-5 (154.3 mg) was injected into the preparative high-performance column chromatography (HPLC) and eluted using a gradient of methanol and de-ionized water (70:30 to 100% MeOH in 45 min), which showed a prominent peak, that produced a single spot labeled as **1** (R_t_ 14.06 min, 19.9 mg, 0.023%).

Sub-fraction XIII-4 (255 mg) was also injected into HPLC under the same condition, which showed a prominent peak, that afforded a single spot labeled as **2** (R_t_ 27.09 min, 11.2 mg, 0.013%).

Main fraction XIV (1077 mg) was applied to repeated silica gel column under the same condition. Sub-fraction SA-XIV-2 (694.4 mg) was injected to the HPLC under the same condition, which showed two prominent peaks, that afforded a single spot each and were labeled as **3** (R_t_ 19.65 min, 7.1 mg, 0.023%) and **5** (R_t_ 27.41 min, 7.2 mg, 0.0084%).

Main fraction XI (1504.3 mg) was also applied to a repeated silica gel column under the same condition. Fractions 16–22 showed only a single spot and the compound was labelled as **4** (15.2 mg, 0.017%).

### 2.4. Spectroscopic Data of the Isolated Compounds

**Compound 1**. ^1^H NMR (400 MHz, CDCl_3_), δ_H_ 6.64 (*s*, H-14), 5.38 (*dd*, H-7, *J* = 1.3; 1.3 Hz), 3.08 (*sept*, H-15, *J* = 6.9 Hz), 2.91 (*br d*, H-1α, *J* = 12.8 Hz), 2.40 (*ddd*, H-1β, *J* = 4.32, 13.2, 13.2 Hz), 1.88 (*ddd*, H-6, *J* = 1.64; 10.8; 10.8 Hz), 1.87 (*dddd*, H-2α, *J* = 1.4, 10.6, 10.6, 10.6 Hz), 1.60 (*m*, H-2β), 1.6 (*s*, H-5), 1.32 (*d*, H-3β, *J* = 14.1 Hz), 1.3 (*dd*, H-3α, *J* = 3.8, 13.6 Hz), 1.24 (*d*, Me-17, *J* = 6.9 Hz), 1.22 (*d*, Me-16, *J* = 6.9 Hz), 0.90 (*s*, Me-19), 0.86 (*s*, Me-18). ^13^C NMR (100 MHz, CDCl_3_), δ_C_ 175.8 (C-20), 141.7 (C-12), 141.1 (C-11), 132.8 (C-13), 132.1 (C-8), 121.6 (C-9), 112.3 (C-14), 77.9 (C-7), 45.4 (C-5), 48.4, (C-10), 41.0 (C-3), 34.5 (C-4), 31.7 (C-18), 29.7 (C-1), 29.2 (C-6), 27.3 (C-15), 22.5 (C-17), 22.4 (C-16), 19.7 (C-19), 18.9 (C-2).

**Compound 2**. ^1^H NMR (400 MHz, CDCl_3_), δ_H_ 6.8 (*s*, H-14), 4.67 (*d*, H-7, *J* = 3.0 Hz), 4.49 (*d*, H-6, *J* = 2.7 Hz), 3.09 (*br d*, H-1β, *J* = 14.2 Hz), 3.01 (*sept*, H-15, *J* = 6.3 Hz), 2.1 (*s*, H-5), 1.97 (*ddd*, H-1β, *J* = 4.4, 13.2, 13.2 Hz), 1.60 (m, H-2α), 1.47 (m, H-2β), 1.41 (*br d*, H-3, *J* = 12.5 Hz), 1.27 (*m*, H-3), 1.14 (*d*, Me-16, *J* = 6.2 Hz), 1.15 (*d*, Me-17, *J* = 6.2 Hz), 0.94 (*s*, Me-18), 0.84 (*s*, Me-19). ^13^C NMR (100 MHz, CDCl_3_), δ_C_ 178.8 (C-20), 142.7 (C-11), 141.8 (C-12), 135.0 (C-13), 128.0 (C-8), 124.4 (C-9), 120.2 (C-14), 78.1 (C-6), 68.4 (C-7), 50.7 (C-5), 47.1 (C-10), 38.1 (C-3), 31.5 (C-18), 31.4 (C-4), 29.3 (C-1), 27.3 (C-15), 22.5 (C-17), 22.2 (C-19), 22.0 (C-16), 19.0 (C-2).

**Compound 3**. ^1^H NMR (400 MHz, CDCl_3_), δ_H_ 6.8 (*s*, H-14), 4.71 (*d*, H-7, *J* = 3.0 Hz), 4.26 (*d*, H-6, *J* = 3.0 Hz), 3.66 (*s*, OCH_3_), 3.48 (*s*, H-5), 3.15 (*br d*, H-1β, *J* = 14.3 Hz), 3.07 (*sept*, H-15, *J* = 6.8 Hz), 2.0 (*ddd*, H-1α, *J* = 5.3, 5.3, 13.6 Hz), 1.67 (*m*, H-2α), 1.56 (*m*, H-2β), 1.45 (*br d*, H-3β, *J* = 12.5 Hz), 1.23 (*d*, H-3α, *J* = 1.6 Hz), 1.24 (*d*, Me-16, *J* = 6.8 Hz), 1.22 (*d*, Me-17, *J* = 6.8 Hz), 1.01 (*s*, Me-18), 0.93 (*s*, Me-19). ^13^C NMR (100 MHz, CDCl_3_), δ_C_ 179.0 (C-20), 142.6 (C-11), 141.5 (C-12), 134.6 (C-13), 126.4 (C-8), 124.5 (C-9), 120.8 (C-14), 77.4 (C-6), 74.7 (C-7), 58.3 (OCH_3_)_,_ 50.8 (C-5), 47.1 (C-10), 38.0 (C-3), 31.6 (C-18), 31.3 (C-4), 27.2 (C-1), 27.1 (C-15), 23.5 (C-17), 22.2 (C-16), 22.0 (C-19), 19.0 (C-2).

**Compound 4**. ^1^H NMR (400 MHz, CDCl_3_), δ_H_ 6.44 (*s*, H-14), 3.66 (*s*, OCH_3_), 3.45 (*br d*, H-1β, *J* = 13.2 Hz), 3.09 (*sept*, H-15, *J* = 6.8 Hz), 2.76 (*m*, H-7), 2.25 (*ddd*, H-5, *J* = 6.9, 12.5, 12.5 Hz), 1.77 (H-6), 1.75 (*br d*, H-2α, *J* = 12.8 Hz), 1.48 (*d*, H-2β, *J* = 14.2 Hz), 1.43 (*d*, H-3α, *J* = 2.9 Hz), 1.24 (*d*, H-3β, *J* = 4.5 Hz), 1.17 (*d*, H-1α, *J* = 5.8 Hz), 1.14 (*d*, H-17, *J* = 6.8 Hz), 1.12 (*d*, H-16, *J* = 6.8 Hz), 0.89 (*s*, H-18), 0.79 (*s*, H-19). ^13^C NMR (100 MHz, CDCl_3_), δ_C_ 181.0 (C-20), 147.8 (C-11), 142.3 (C-12), 139.5 (C-13), 134.5 (C-8), 125.3 (C-9), 118.1 (C-14), 61.7 (OCH_3_), 47.7 (C-10), 41.5 (C-5), 41.5 (C-3), 34.1 (C-1), 34.1 (C-4), 31.9 (C-7), 26.5 (C-15), 23.8 (C-17), 23.5 (C-16), 20.0 (C-19), 19.9 (C-2).

**Compound 5**. ^1^H NMR (400 MHz, CDCl_3_), δH 12.82 (*s*, 5-OH), 7.85 (*d*, *J* = 8.9 Hz, H-2′, H-4′), 7.01 (*d*, *J* = 8.9 Hz, H-1′, H-6′), 6.58 (*s*, H-3), 6.49 (*d*, *J* = 2.2 Hz, H-6), 6.37 (*d*, *J* = 2.2 Hz, H-8), 3.89 (*d*, *J* = 4.7 Hz, 6H, 2X ArOCH3).

### 2.5. Alpha-Glucosidase Inhibitory Activity

The alpha-glucosidase assay of the tested compounds (**1**–**5**) was conducted according to the standard method with slight modification [22]. Inside the 96-well plate, 50 μL of phosphate buffer (100 mM, pH = 6. 8), 10 μL alpha-glucosidase (1 U/mL), 20 µL of samples, and standard (acarbose) of different concentration were incubated for 15 min at 37 °C. Briefly, 20 μL of 5 mM substrate (4-nitrophenyl β-d-glucopyranoside) was added to each well and left to incubate for 20 min at 37 °C. The reacting mixture was stopped after incubation by adding 0.1 M sodium carbonate (50 μL). The release of p-nitrophenol of the reacting mixture relating to the activity of the enzyme was read at a wavelength of 405 nm using a multiplate reader (Multiskan, Thermo Scientific). The result represents the mean of three independent experiments and is expressed as percentage inhibition calculated as stated below:

Percentage inhibitory activity (%) = (1 − A/B) × 100

where, A is the absorbance in the presence of test substance, and B is the absorbance in the presence of phosphate buffer (control).

### 2.6. Alpha-Amylase Inhibitory Activity

The alpha-amylase assay of the tested compounds (**1**–**5**) was conducted according to the standard method with slight modification [22]. In a 96-well plate, the reaction mixture containing 50 μL of phosphate buffer (100 mM, pH = 6.8), 10 μL alpha-amylase (2 U/mL), 20 μL of varying concentrations of sample (100–31.2 μg/mL), and standard. The mixture was allowed to incubate for 20 min at 37 °C before the addition of 1% soluble starch (10 μL) and further incubated for another 30 min. A color reagent DNS (100 μL) was added and boiled at 95 °C for 15 min. The change in color was read at a wavelength of 450 nm using a multiplate reader. The results are the mean of three independent studies and calculated as percentage inhibition as stated below:

Percentage inhibitory activity (%) = (1 − A/B) × 100

where, A is the absorbance in the presence of test substance, and B is the absorbance in the presence of phosphate buffer (control).

### 2.7. Molecular Docking Analysis

#### 2.7.1. Selection and Preparation of Ligands

The chemical structures of five pure compounds were obtained from the PubChem compound database (https://pubchem.ncbi.nlm.nih.gov). The Molfiles Structure Data File (MOL SDF) formats of these compounds were converted to Protein Data Bank, Partial Charge (Q), and Atom Type (T) (PDBQT) file using the PyRx tool to generate atomic coordinates, and energy was minimized by optimization using the optimization algorithm at force field set at mmff94 (required) on PyRx.

#### 2.7.2. Retrieval and Preparation of Alpha-Amylase and Alpha-Glucosidase Drug Target

The three-dimensional crystal structure of alpha-amylase Protein Data Bank (PDB) ID: 3BAI and resolution 1.9 Å) and alpha-glucosidase (PDB ID: 2QMJ) in complex with co-crystallized ligand (acarbose), an inhibitor of both proteins was retrieved from Research Collaboratory for Structural Bioinformatics (RCSB) PDB (http://www.rcsb.org/pdb/home/home.do). The proteins were prepared using PyMol tool. While preparing the proteins, the bound complex molecules with the proteins and non-essential water molecules were removed. Moreover, Discovery Studio 2017 R2 was employed to eliminate entire heteroatoms. The co-crystallized ligand was extracted from the protein’s active site so as to reveal the grid coordinate around the binding pocket when viewed on PyMol and Discovery Studio 2017 R2 visualizer.

#### 2.7.3. Preparation of the Standards

Co-crystalized ligand acarbose of alpha-amylase and alpha-glucosidase was used as standard in this study. The chemical structure of the co-crystalized ligand (PDB Ligand ID: ACR) extracted from the enzymes’ active sites was converted to a PDBQT file using the PyRx tool to generate atomic coordinates, and energy was minimized by optimization using the optimization algorithm at force field set at mmff94 (required) on PyRx.

#### 2.7.4. Molecular Docking Using PyRx

Following drug target and ligand preparation, molecular docking analysis was performed using PyRx, AutoDock Vina option based on scoring functions. After the docking process, the grid box resolution was centered at 8.3860 × 28.6135 × 50.1812 for alpha-amylase while it was −29.930 × 7.7002 × −17.8920 for alpha-glucosidase along the x, y, and z axes, respectively, at a grid dimension of 25 × 25 × 25 Å to define the binding site. The standards were first docked within the binding site of the enzymes and the resulting interactions were compared with that of isolated compounds into the same active sites using the same grid box dimension.

#### 2.7.5. Validation of Docking Protocol

Validation of docking protocol accuracy was done by redocking the co-crystallized ligand back into the binding site of alpha-glucosidase. Docking methodology was found to be reliable as the re-docked pose overlapped almost totally with the experimental orientation. This indicates that AutoDock Vina on PyRx re-docked the co-crystallized ligand, with a very high accuracy, back into the binding pocket of alpha-amylase and alpha-glucosidase.

### 2.8. Antioxidant Assays

#### 2.8.1. Ferric-Ion Reducing Antioxidant Power (FRAP) Assay

The FRAP assay was assessed according to the method of Benzie and Strain (1996) [23]. FRAP reagent containing the mixture of acetate buffer (300 mM, pH 3.6), tripyridyl triazine (TPTZ) (10 mM in 40 mM HCl), and 20 mM FeCl_3_.6H_2_O in ratio 10:1:1 (v/v/v) was used. Extract, compounds, or standard (10 µL) was added to 300 µL FRAP reagent, incubated for 30 min in the dark at room temperature. The reacting mixture was read at a wavelength of 593 nm in a multiplate reader. Ascorbic acid was used as standard at varying concentrations of 0 to 1000 µM. The result was presented as a mean of independent triplicate experiments and expressed as µM ascorbic acid equivalents per milligram dry weight (µM AAE/g) of the test samples.

#### 2.8.2. Automated Oxygen Radical Absorbance Capacity (ORAC) Assay

The method of Cao and Prior (1998) was employed to measure oxygen radical absorbance capacity (ORAC) and as reported in our previous study [24]. The method measures the scavenging potential of compounds against the decomposition of peroxyl radical of 2,2-azobis (2-amino-propane) dihydrochloride (AAPH) as peroxyradical (ORAC _ROO_^•^). The antioxidant capacity of a compound is a measure of the fading fluorescence of a probe (fluorescein) using the area under the curve (AUC) plot in relation to a control blank. The florescence of the probe was programmed to be measured at every two minutes for 2 h after the addition of AAPH at the excitation wavelength and emission set at 485 and 530 nm, respectively. The ORAC results were estimated following a regression equation (Y = a + bX + Cx^2^) between Trolox concentration (Y in μM) and the net area under the fluorescence decay curve (X). ORAC values were expressed as μM Trolox equivalent (TE)/mg of test sample.

#### 2.8.3. Trolox Equivalent Absorbance Capacity (TEAC) Assay

The TEAC assay was evaluated following the method of Pellegrini et al. (1999) [25]. The working solution containing 88 µL of K_2_S_2_O_8_ (140 mM) and 5 mL ABTS (7 mM) was kept for at least 16 h in the dark at 25 °C. The working solution was then diluted after 16 h with ethanol until the absorbance read approximately 2.0 (±0.1). The extract, purified compounds, or standard (25 µL) was mixed with 300 µL working solution and allowed to incubate in the dark for 30 min at room temperature. Trolox was used as the standard using a concentration range between 0 and 500 µM. The absorbance of the reaction was read at a wavelength of 734 nm using a multiplate reader.

### 2.9. Statistical Analysis

All the measurements were repeated three times, and the IC_50_ was calculated using GraphPad Prism 5 version 5.01 (Graph pad software, Inc., La Jolla, CA, USA) statistical software. The data presented are means ± SD obtained from 96-well-plate readers for all in vitro experiments.

## 3. Results and Discussion

### 3.1. Chemical Characterization

**Figure 1:**
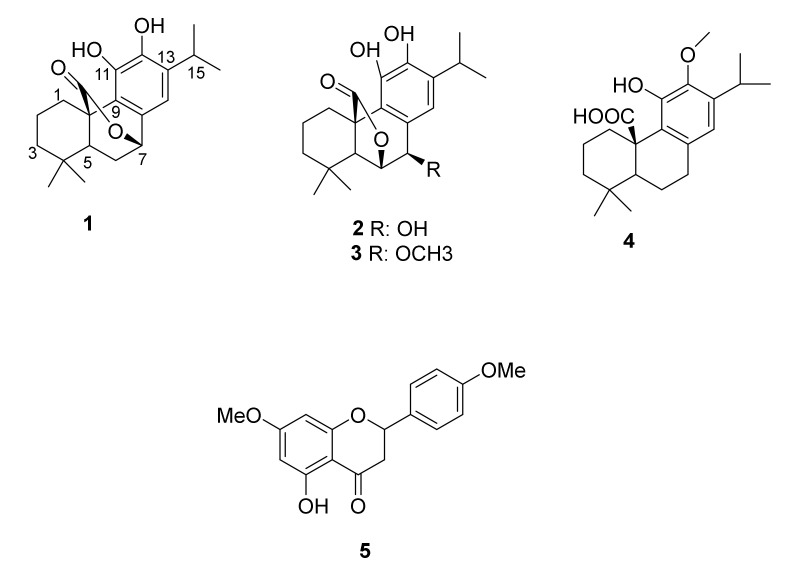
Chemical structures of the isolated compounds (**1**–**5**) from *Salvia aurita*.

Repeated silica gel column chromatography and Prep-HPLC of a methanolic extract of *S. aurita* led to the isolation of four pure terpenoids and one flavonoid (Figure 1).

### 3.2. Biological Evaluation

#### 3.2.1. Alpha-Glucosidase and Alpha-Amylase Activities

The main enzymes involved in the digestion of carbohydrates are alpha-glucosidase and alpha-amylase, together with lipids [26]. Their mechanism of action involves the breakdown of carbohydrates by alpha-amylase, while alpha-glucosidase breaks down starch and disaccharides to glucose [26,27]. One of the therapeutic ways to combat DM is to delay postprandial hyperglycemia by reducing the absorption of glucose through the inhibition of carbohydrate-hydrolyzing enzymes (alpha-amylase and alpha-glucosidase) in the gastrointestinal canal [28,29]. Therefore, suppressors of these enzymes delay carbohydrate digestion, which causes a reduction in the rate of glucose absorption and, consequently, reduces the postprandial increase of plasma glucose [30]. Hence, many efforts have been made to look for more effective and safe inhibitors of alpha-glucosidase and alpha-amylase from natural sources for the development of physiologically functional drugs for the prevention and management of diabetes [31]. The in vitro bio-evaluation of *S. aurita* against alpha-glucosidase and alpha-amylase was performed, and the results demonstrated that **3** exhibited the highest alpha-glucosidase inhibitory capacity with an IC_50_ value of 4.02 ± 0.7 µg/mL, followed by **2** and **5** with IC_50_ values of 15.96 ± 1.0 and 28.74 ± 0.9 µg/mL, respectively, while **4** displayed the highest alpha amylase inhibitory activity with an IC_50_ value of 16.2 ± 0.3 µg/mL followed by **1** and **2** with IC_50_ values of 19.8 ± 1.4 and 40.9 ± 1.2 µg/mL, respectively, as showed in Table 1.

Carnosol (**1**) has been reported to demonstrate significant antidiabetic activities in different models with different mechanisms of action. It increases skeletal muscle cell glucose uptake via 5’ adenosine monophosphate-activated protein kinase (AMPK)-dependent GLUT4 glucose transporter translocation, which is targeted for glucose homeostasis, meaning that it could be a potential antidiabetic compound. Furthermore, structural features of the abietane diterpenes reported to have antidiabetic activity contain COOH groups, lactone rings, and steroid type structures [32]. Carnosol also possesses hypoglycemic and antihyperlipidemic activities, and it has considerable protective effects on the liver and renal functions in diabetic rats [33] as well as ameliorating diabetes complications by modulating oxidative stress [34]. Additionally, carnosol can deactivate intracellular de novo triglyceride synthesis by 67.5–90.6% without affecting cell viability [35] and possesses powerful inhibitory activity with an IC_50_ value of 62.5 µM against rat liver diacylglycerol acyltransferase 1 (DGAT1), an enzyme responsible for triglyceride synthesis [35]. Moreover, 7-*O*-methylrosmanol (**3**) has been reported to diminish forskolin (FSK)-induced luciferase expression when monitored by cAMP/response element (CRE) [36]. Hence, the suppression of cAMP/protein kinase response of the *PEPCK-C* or *G6Pase* gene may add to the antihyperglycemic activity, which is one of the keys in the management of diabetes [36]. The alpha-glucosidase inhibitory activity demonstrated by **1** and **3** are in agreement with some published data reporting on different mechanisms of action exhibited by these compounds [34,36].

#### 3.2.2. Molecular Docking

**Figure 2:**
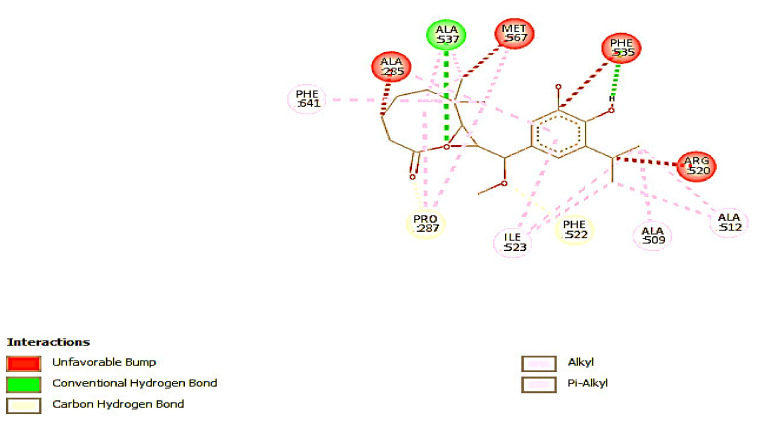
2D interaction of 7-methoxyrosmanol with amino acid residues of alpha-glucosidase.

**Figure 3:**
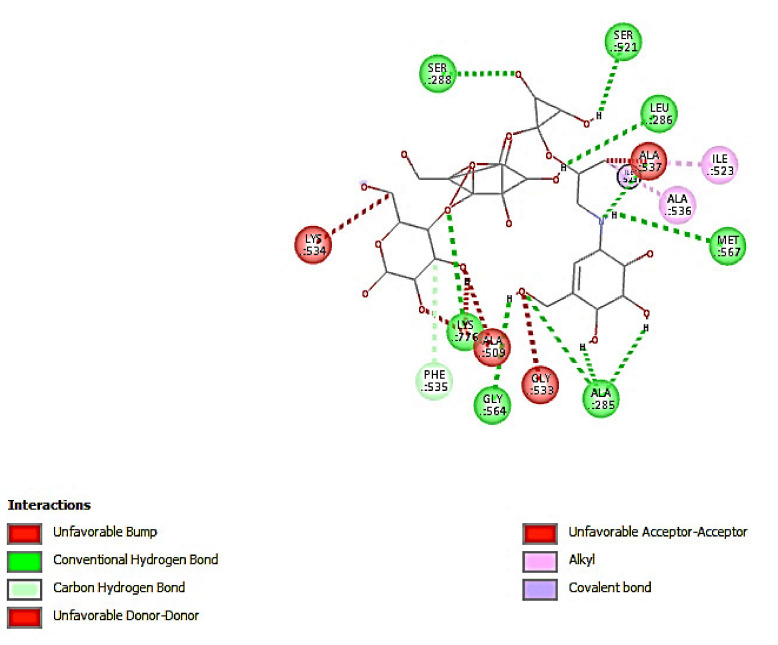
Interaction of acarbose with amino acid residues of alpha-glucosidase.

Molecular docking analysis shows that there is no leading compound among the isolated compounds docked against alpha-amylase. All the compounds have binding energy lower than standard acarbose with −8.5 Kcal/mol (Table 2). The result is in accord with the observed in vitro experiment with acarbose having the lowest IC_50_ compared with that of other compounds. Contrarily, however, the docking results of alpha-glucosidase show that 7-methoxyrosmanol is the leading compound with the highest binding energy of −14.9 Kcal/mol while standard had −14.5 Kcal/mol. This result is in consonance with what is obtained in the in vitro experiment, with 7-methoxyrosmanol having the lowest IC_50_ value, which implies the highest inhibitory activity as compared to that of other compounds. The docking complex of 7-methoxyrosmanol with alpha-glucosidase was stabilized by four hydrogen bonds and fourteen hydrophobic interactions as presented in Table 3 and Figure 2. Acarbose on the other hand had twelve hydrogen bonds and three hydrophobic interactions (Table 4 and Figure 3). The amino residues of alpha-glucosidase that are common to both acarbose and 7-methoxyrosmanol in providing hydrogen bonds are ALA 537 and PHE 535 with a distance of 2.75117 and 3.11041 Å, respectively, for acarbose and 2.97128 and 3.03951 Å, respectively, for 7-methoxyrosmanol. In addition, ALA 536, ALA 537, and ILE 523 residues that provide hydrophobic interaction for acarbose are also part of the residues involved in 7-methoxyrosmanol hydrophobic interactions. This indicates that 7-methoxyrosmanol binds to alpha-glucosidase in a space close to where the acarbose binds to the protein. Acarbose is a known competitive inhibitor of alpha-glucosidase, which means that it binds to the active site of the protein [37]. This shows that 7-methoxyrosmanol might also be a competitive inhibitor of alpha-glucosidase.

#### 3.2.3. Antioxidant Activity

Diabetes mellitus involves oxidative stress in both etiology and pathogenesis pathways. However, oxidative stress happens when the generation of free radicals such as reactive oxygen species (ROS), including superoxide radical, hydrogen peroxide, and hydroxyl radical, overpowers the inherent cellular antioxidant defense system leading to oxidative stress and eventual damage to macromolecules related to the release of insulin [38]. Oxidative stress has been implicated to play pivotal roles in the development of several disease complications such as microvascular and cardiovascular diabetes complication [39]. The disruption of physiological metabolic process by diabetes causes leakage and overproduction of superoxide anion in mitochondrial energy metabolism. The excessive production and seeping of superoxide anion is essential for diabetes complications such as insulin resistance, β-cell dysfunction, and weakened glucose tolerance [40]. The usage of medicinal herbs in the management of diabetes and oxidative stress is well known, and these herbs contain bioactive constituents such as flavonoids, polyphenols, alkaloids, terpenoids, carotenoids, vitamins, and numerous other phytochemicals, which can act as antidiabetics and/or antioxidants/radical scavengers [41].

Abietane type diterpenes are well reputed to exhibit powerful radical scavenging activity because of the existence of ortho-dihydroxyl groups in the benzene ring, serving as hydrogen atom abstracting and/or electron donating agents and metal ion-chelators [42]. Additionally, the presence of the ortho-dihydroxyl group at the aromatic ring in the abietane diterpenes is responsible for the prominent biological activities displayed by these groups of compounds [43]. Phenolic compounds are types of natural phytochemicals that play a dual role as antioxidant and prooxidant. Antioxidant property is beneficial because of the ability to scavenge free radicals, while prooxidation promotes the oxidation reaction, which is lethal. The phenoxyl radical produced from phenolic compounds can be soothed by either addition of protons or loss of electrons to another molecule [44].

The antioxidant activity of the isolated compounds of the methanolic extract of *S. aurita* was investigated by assessing their FRAP, TEAC, and ORAC activities. The results demonstrated excellent antioxidant activity for **2**, **1,** and **3,** respectively, with ORAC (25.79 ± 0.01; 23.96 ± 0.01; 23.94 ± 0.02) mM TE/g; **1** and **2** as FRAP (3.92 ± 0.002; 1.52 ± 0.002) mM AAE/g; and **5** and **2** as TEAC (3.19 ± 0.003; 2.06 ± 0.003) mM TE/g. The crude extract of *S. aurita* exhibited moderate antioxidant activity when tested on ORAC (4.45 ± 0.01 mM TE/g), TEAC (723.9 ± 6.4 mM TE/g), and FRAP (393.7 ± 2.3 mM AAE/g) as showed in Table 5. The antioxidant prowess of the isolated compounds are comparable with the standard (EGCG) used in this study with FRAP (7.53 ± 0.005 mM AAE/g), TEAC (4.15 ± 0.02 mM TE/g), and ORAC (3.98 ± 0.00 mM TE/g). Therefore, the activity of **2** and **1** is associated to the presence of the ortho-dihydroxyl groups located on the aromatic ring, serving as hydrogen atom transferring agent to peroxyl radicals, whereby, stabilizing/neutralizing them and inducing to a stable radical [45]. Additionally, carnosol and rosmanol share a common chemical structure framework, which may possibly increase their bioactivity. Our findings also indicated that the higher the number of hydroxyl groups in these phenolic compounds, the higher the antioxidant activity is. The highest bio-activity demonstrated by rosmanol can then be justified by the fact that it contains three free hydroxyl groups compared to carnosol that has only two. Rosmanol and 7-methoxyl rosmanol share the same chemical structure framework, but the difference in the activity exhibited should be explain by the fact that the substitution of the hydroxyl group in 7-methoxyl rosmanol might be responsible of the decrease of the activity observed. The structure–activity relationship (SAR) displayed that the substitution of the hydroxyl group at C-7 by the methoxyl group is directly linked to the bioactivity demonstrated. Although, the antioxidant capacity of carnosol is high because of the highly stressed lactone ring, which might be opened during the process of chemical reaction, leading to a rise in conjugation and formation of *p*-quininoidal structure. In 7-methoxyrosmanol, antioxidant activity is less compared to that in carnosol and rosmanol, possibly due to the presence of a methoxyl group at position C-7, which is responsible for the hydrophobicity characteristic in the compounds.

Carnosol, carnosic acid, and rosmanol are the most abundant phytochemical constituents of *Rosmarinus officinalis* and *Salvia* species, whereby they contribute to approximately 90% of the total antioxidant capacity of Lamiaceae [9]. Rosmanol and carnosol have been reported to display significant antioxidant capacity, in a competitive manner with tocopherol [18,19]. These reported data corroborate with our findings.

## 4. Conclusions

The phytochemical and biological investigations of the methanolic extract of *S. aurita* disclosed that this plant is a rich source of abietane diterpenes with strong alpha-glucosidase and alpha-amylase inhibitory activities as well as excellent antioxidant capacities. This is the first scientific report on the phytochemical isolation and biological evaluation of alpha-glucosidase and alpha-amylase inhibitory activities of *S. aurita,* and the results possibly suggest that the methanolic extract of *S. aurita* and/or its individual isolated compounds might become notable natural therapeutic candidates against alpha-glucosidase and alpha-amylase enzymes and oxidative stress. Therefore, compounds that demonstrate remarkable alpha-glucosidase and alpha-amylase inhibitory and antioxidant capacities might be very good candidates for controlling the oxidative stress, plasma glucose level and accompany complications in diabetic patients.

## Figures and Tables

**Table 1:** Inhibitory activities of *S. aurita* constituents on alpha-glucosidase and alpha-amylase.

Items	Alpha-Glucosidase IC_50_ (µg/mL)	Alpha-Amylase IC_50_ (µg/mL)
Carnosol	51.8 ± 1.9	19.8 ± 1.4
Rosmanol	16.4 ± 1.4	40.9 ± 1.2
7-methoxyrosmanol	4.2 ± 0.7	NA
12-methoxycarnosic acid	36.9 ± 2.1	16.2 ± 0.3
4,7-dimethylapigenin ether	28.7 ± 0.9	NA
Crude extract	241.9 ± 2.7	NA
Acarbose	610.4 ± 1.0	10.2 ± 0.6

The results are expressed as mean ± SD for *n* = 3. Acarbose is used as standard for the experiments. NA: not active within the evaluated concentrations.

**Table 2:** Binding energy of interactions between compounds and enzymes (alpha-amylase and alpha-glucosidase).

S/N	Compounds	PubChem CID	α-Amylase Binding Energy (Kcal/mol)	α-Glucosidase Binding Energy (Kcal/mol)
1	Carnosol	442,009 (1)	−6.3	−5.6
2	Rosmanol	13,966,122	−5.2	−6.9
3	7-methoxyrosmanol	9,950,773	−6.4	−14.9
4	12-methoxycarnosic acid	9,974,918	−6.4	−5.6
5	4,7-dimethyl apigenin	5,281,601	−6.1	−4.3
6	Acarbose (standard)	41,774	−8.5	−14.5

**Table 3:** Hydrogen and hydrophobic interactions of 7-methoxyrosmanol and alpha-glucosidase.

NAME	CATEGORY	DISTANCE (Å)
.:ALA537:HNN: N: 7-methoxyrosmanol:O	Hydrogen Bond	2.97128
N: 7-methoxyrosmanol:H—.:PHE535:O	Hydrogen Bond	3.03951
.:PRO287:CA—N: 7-methoxyrosmanol:O	Hydrogen Bond	2.90164
.:PHE522:CA—N: 7-methoxyrosmanol:O	Hydrogen Bond	2.85006
.:PRO287—N: 7-methoxyrosmanol	Hydrophobic	5.06789
.:ALA509—N: 7-methoxyrosmanol:C	Hydrophobic	4.05099
.:ALA512—N: 7-methoxyrosmanol:C	Hydrophobic	3.63559
.:ALA512—N: 7-methoxyrosmanol:C	Hydrophobic	3.80942
.:ALA537—N: 7-methoxyrosmanol	Hydrophobic	5.1986
.:ALA537—N: 7-methoxyrosmanol:C	Hydrophobic	3.12883
.:MET567—N: 7-methoxyrosmanol	Hydrophobic	5.24033
N: 7-methoxyrosmanol:C—.:PRO287	Hydrophobic	3.6525
N: 7-methoxyrosmanol:C—.:MET567	Hydrophobic	2.66937
N: 7-methoxyrosmanol:C—.:ILE523	Hydrophobic	5.18761
N: 7-methoxyrosmanol:C—.:ILE523	Hydrophobic	4.28153
.:PHE641—N: 7-methoxyrosmanol:C	Hydrophobic	4.60588
N: 7-methoxyrosmanol—.:ALA285	Hydrophobic	4.16732
N: 7-methoxyrosmanol—.:ILE523	Hydrophobic	4.57178

**Table 4:** Hydrogen and hydrophobic Interactions of acarbose and alpha-glucosidase.

NAME	CATEGORY	DISTANCE (Å)
.:ALA285:HN—N:Acarbose:O	Hydrogen Bond	3.08738
.:SER288:HN—N: Acarbose:O	Hydrogen Bond	2.65592
.:ALA537:HN—N: Acarbose:N	Hydrogen Bond	2.75117
.:LYS776:HZ1—N: Acarbose:O	Hydrogen Bond	2.43057
N: Acarbose:H—.:MET567:SD	Hydrogen Bond	2.65026
N: Acarbose:H—.:ALA285:O	Hydrogen Bond	2.41018
N: Acarbose:H—.:ALA285:O	Hydrogen Bond	2.89448
N: Acarbose:H—.:GLY564:O	Hydrogen Bond	2.20166
N: Acarbose:H—.:SER521:O	Hydrogen Bond	2.51361
N: Acarbose:H—.:LEU286:O	Hydrogen Bond	2.23783
N: Acarbose:H—N:UNK1:O	Hydrogen Bond	3.04118
N: Acarbose:C—.:PHE535:O	Hydrogen Bond	3.11041
.:ALA536—N: Acarbose:C	Hydrophobic	3.29491
.:ALA537—N: Acarbose:C	Hydrophobic	3.37906
N: Acarbose:C—.:ILE523	Hydrophobic	4.52239

**Table 5:** Total antioxidant capacity of *S. aurita* constituents.

Items	ORAC (mM TE/g)	TEAC (mM TE/g)	FRAP (mM AAE/g)
Carnosol	23.96 ± 0.01	0.33 ± 0.002	3.92 ± 0.002
Rosmanol	25.79 ± 0.01	2.06 ± 0.003	1.52 ± 0.002
7-methoxyrosmanol	23.94 ± 0.02	0.22 ±0.002	1.32 ± 0.002
12-methoxycarnosic acid	20.25 ± 0.01	0.34 ± 0.003	0.51 ± 0.003
4,7-dimethylapigenin ether	6.47 ± 0.01	3.19 ± 0.003	0.61 ± 0.006
Crude extract	4.45 ± 0.00	0.72 ± 0.006	0.39 ± 0.002
EGCG	3.98 ± 0.00	4.15 ± 0.02	7.53 ± 0.005

The results are expressed as Mean ± SD of triplicate experiments. EGCG (epigallocatechin-3-gallate) is used as standard. TE (Trolox equivalent); AAE (Ascorbic acid equivalent). ORAC, oxygen radical absorbance capacity; TEAC, Trolox equivalent absorbance capacity; FRAP, ferric-ion reducing antioxidant power.
